# Psychosocial Risks and Protective Factors for Healthcare Worker Burnout During the Post-Acute Phase of the COVID-19 Pandemic

**DOI:** 10.3390/ejihpe15090186

**Published:** 2025-09-16

**Authors:** Kristīne Dūdiņa, Baiba Martinsone

**Affiliations:** 1 Department of Psychology, University of Latvia, LV-1004 Riga, Latvia; kristine.dudina@lu.lv; 2 Department of Neuromedicine and Neuroscience, University of Latvia, LV-1004 Riga, Latvia

**Keywords:** burnout, healthcare workers, psychosocial risks, work–life conflict, buffering effect, amplification effect, self-endangering behaviour

## Abstract

Burnout is a critical problem among healthcare professionals worldwide, but nationally representative data on psychosocial factors associated with burnout are lacking for Latvia’s hospital system. This study investigated twofold aims: first, it examined the association between job-related demands, psychosocial resources, and burnout in a representative sample of Latvian hospital staff; and second, it tested whether specific resources buffer or amplify the impact of excessive workload. A cross-sectional survey was conducted among 4756 healthcare workers across 30 inpatient institutions in Latvia. Participants completed the Copenhagen Psychosocial Questionnaire III and the Burnout Assessment Tool; regression and moderation analyses were used. Burnout was positively associated with longer working hours, multiple job-holding, and psychosocial demands such as emotional strain, time pressure, and work–life conflict. Several resources, including support from colleagues, supervisor support, recognition, sense of belonging, supervisor evaluation, and especially resources for quality work, were associated with lower burnout and weakened the relationship between workload and burnout. In contrast, high autonomy, meaning at work, organizational justice, and role conflict amplified this association. These findings suggest that in resource-constrained healthcare systems, some job resources may be associated with increased risk of burnout. Effective interventions should address both structural and relational factors to mitigate burnout among healthcare workers.

## 1. Introduction

The prevalence of mental health issues among healthcare workers is higher than the average in the general population. Globally, nearly half of healthcare professionals report experiencing burnout, raising serious concerns about workforce well-being and the sustainability of healthcare systems (Luo et al., 2020). Despite increased research and attempts to remedy the strains experienced by healthcare workers, burnout levels remain persistently high, calling for evidence-based, effective strategies (Cohen et al., 2023; Maunder et al., 2022; Wu et al., 2024). Our research contributes to the growing body of evidence on processes driving the burnout pandemic and tentatively suggests possible solutions while also contributing to the main theories of burnout.

Among other mental health problems, burnout is particularly relevant as a work-specific syndrome that reflects the chronic strain experienced by healthcare professionals in demanding environments (De Hert, 2020; Nagle et al., 2024). The World Health Organization’s ICD-11 defines burnout as an occupational phenomenon resulting from chronic workplace stress that has not been successfully managed (World Health Organization, 2019). It is characterized by feelings of energy depletion or exhaustion, increased mental distance from one’s job or feelings of negativism or cynicism related to one’s job, and reduced professional efficacy (Edú-Valsania et al., 2022). Burnout frequently co-occurs with symptoms of depression, anxiety, and various psychosomatic complaints (Bianchi et al., 2021).

Burnout is highly prevalent among healthcare workers, with systematic reviews reporting rates of 30–50% across different settings (Boucher et al., 2025). It became a particularly salient occupational health issue during the COVID-19 pandemic, with studies documenting heightened emotional exhaustion and stress among healthcare professionals (Maunder et al., 2022; Cohen et al., 2023). An umbrella review of 87 meta-analyses found that 44% of healthcare workers experienced clinically significant symptoms during this period (Boucher et al., 2025). In Latvia, no representative data were available before this study; however, a longitudinal survey of general practitioners showed sharp increases in depression and anxiety during the pandemic, with rates peaking at 43% and 37%, respectively (Valaine, 2025). Given the close empirical and conceptual links between burnout and common mental health conditions such as depression, these findings highlight the severe psychological strain facing Latvia’s healthcare workforce.

Burnout has been linked with poorer quality of care, increased medical errors and adverse events, reduced patient satisfaction, and impaired communication (Li et al., 2024; West et al., 2016). Burnout also contributes to organizational dysfunction by increasing absenteeism, job turnover, and the intention to leave the profession (Özkan, 2022). Healthcare workers experiencing burnout may reduce their working hours or exit the field entirely, further exacerbating workforce shortages. Alleviating provider burnout is thus critical not only for the well-being of clinicians but also for maintaining safe, effective, and sustainable patient care.

Mental health outcomes among healthcare professionals are shaped by both individual vulnerability and systemic job conditions, with psychosocial risks playing a central role in burnout (European Agency for Safety and Health at Work, 2022). The Job Demands–Resources (JD-R) model attributes burnout to high demands unbalanced by resources such as autonomy or social support (Bakker & Demerouti, 2017). The Job Demand–Control–Support (JDCS) model emphasizes that high psychological demands, limited control, and insufficient support intensify risk (Christiansen et al., 2024). The Person–Job Mismatch model further highlights how misalignment between worker expectations and job realities across domains like control, reward, and fairness undermines engagement and identity (Leiter & Maslach, 2003). Collectively, these frameworks highlight the role of excessive workload, emotional demands, role conflict, and inadequate support in shaping burnout among healthcare professionals, factors that are especially pertinent in healthcare, where heavy workloads, time pressure, emotional labour, and exposure to suffering are routine (Gynning et al., 2024).

In Latvia’s predominantly publicly funded system, persistent workforce shortages have left staff particularly vulnerable; an issue intensified during the COVID-19 pandemic. National data indicate approximately 48,900 healthcare professionals, with 56.6% employed in hospitals; as of 2021–2022, Latvia reported among the lowest nurse and physician densities in Europe (4.2 nurses and 3.4 physicians per 1000 population, compared to EU averages of 8.5 and 4.2) (OECD & European Observatory on Health Systems and Policies, 2024). Hospital staff therefore face excessive patient loads, extended working hours, and increased psychosocial risk, contributing to severe workload pressure, psychological distress, and high burnout, with negative implications for staff well-being and the sustainability of patient care.

Given these systemic challenges, it is critical to assess whether psychosocial resources can buffer the effects of high demands. While resources such as supportive leadership, team cohesion, and clinical autonomy are often assumed to play this role, empirical evidence is mixed: some studies have found no significant buffering (Fagerlind Ståhl et al., 2018), while others suggest that effects vary across occupational groups (Gynning et al., 2024). This study contributes to the debate by testing moderation effects within the JD-R framework in a high-demand, resource-constrained hospital context, using the Copenhagen Psychosocial Questionnaire (COPSOQ) III to capture key demands (e.g., workload, emotional demands, role conflict, work–life conflict) and resources (e.g., autonomy, social support, recognition, meaning, organizational justice).

Despite extensive research on burnout among healthcare professionals worldwide, no study has yet examined the associations between job-related demands, resources, and burnout in a representative hospital sample in Latvia. To address this gap, the present study investigates these relationships using a nationally representative sample of hospital employees from 30 institutions across the country. This represents the first comprehensive examination of the mental health of healthcare workers in Latvian hospitals, conducted during the post-acute phase of the COVID-19 pandemic, a period marked by systemic stressors and heightened psychosocial risks.

To address these aims, we examined the following research questions (RQ1–RQ3):

- RQ1. To what extent are sociodemographic and job-related characteristics associated with burnout in hospital-based healthcare professionals during the post-acute phase of the COVID-19 pandemic? (Variables include age, gender, profession, number of workplaces, working hours, and presenteeism.)
- RQ2. Among psychosocial work factors assessed by the COPSOQ III, which factors are most strongly associated with burnout? (Factors include emotional demands, time pressure, and role conflict.)
- RQ3.
  (a) Does work–life interference mediate the association between job demands and burnout? (Job demands include emotional demands and time pressure.)
  (b) Do psychosocial resources moderate the association between job demands and burnout? (Resources include supervisor support, organizational justice, and sense of belonging.)

To address these questions, we formulated four theory-driven hypotheses (H1–H4) grounded in established models of occupational stress and burnout:

**H1.**  *Higher job demands (*e.g., *emotional demands, time pressure, and work–life conflict) will be positively associated with higher levels of burnout, after adjusting for sociodemographic and job-related covariates. This hypothesis is informed by the JD-R and COR models, which emphasize the cumulative strain of excessive demands and their depleting effect on emotional resources.*

**H2.**  *Psychosocial job resources (*e.g., *supervisor support, organizational justice, recognition, and autonomy) will be negatively associated with burnout, controlling for sociodemographic and job-related characteristics.*

**H3.**  *Work–life interference will statistically mediate the association between job demands and burnout, such that higher job demands will be associated with greater work–life interference, which in turn will be associated with higher burnout levels. This hypothesis builds on COR theory, which conceptualizes work–life conflict as a form of chronic resource loss.*

**H4.**  *The association between job demands and burnout will be moderated by psychosocial job resources (*e.g., *supervisor support, organizational justice, and sense of belonging), such that the relationship will be weaker among individuals reporting higher levels of these resources. This is consistent with the buffering hypothesis within the JD-R framework.*

## 2. Materials and Methods

### 2.1. Study Design and Sampling

This research was conducted as part of a national project aimed at developing instruments and procedures to monitor psychosocial hazards and the mental health of hospital employees in Latvia during the COVID-19 pandemic. The study design was approved by the Ministry of Health’s ethical board.

A pilot study was conducted from 7 March to 16 March 2022, in two hospitals in Latvia as part of the development and adaptation of the mental health monitoring instrument for healthcare workers. The pilot aimed to evaluate the clarity, relevance, and feasibility of the survey materials, including the psychosocial work factors and burnout measures. A total of 129 healthcare employees participated in the pilot study (123 women and 6 men), representing a range of clinical and support roles. Feedback from the pilot was used to refine item wording and improve the usability of the electronic and paper-based versions of the survey. All compulsory items from the COPSOQ III short version were included, and additional items and dimensions were selected based on their theoretical relevance and predictive value for burnout. Psychometric properties from the pilot data were used to determine whether the constructs were retained as validated scales or standalone items.

Data collection for the research took place between 16 May and 15 June 2022, in inpatient medical institutions across Latvia, including the Emergency Medical Service (NMPD), with the goal of assessing employees’ psycho-emotional health and their perceptions of various psychosocial factors in the workplace. All participating institutions were general inpatient hospitals, including regional and specialized care facilities; long-term care facilities (e.g., elderly homes), outpatient clinics, and psychiatric hospitals were not included in this sample. This study was approved by the Ethics Committee of the National Health Service of Latvia (Contract No. NVD-9/120-2021, 21 September 2021). The survey was administered in two formats: as an online questionnaire distributed electronically via internal hospital systems, and as a printed version made available in paper format in hospitals where digital access was limited. Participation was anonymous, the survey was conducted anonymously, no personally identifiable information was collected, and all data were analyzed in aggregate form only. Each hospital appointed a local coordinator responsible for overseeing data collection on-site, following strict guidelines provided by the research coordinator to ensure consistency and standardization across all locations.

A census-style sampling strategy was employed, inviting all eligible hospital employees across participating institutions to partake in the study. Over a three-week data collection period, participants received three invitation emails and were encouraged to participate by their supervisors and, in some instances, hospital managers. All participants provided informed consent prior to participation and were informed that their responses would remain anonymous and that they could withdraw from the study at any time without any consequences.

A total of 5142 healthcare professionals from 30 inpatient facilities (out of 37 governmental inpatient facilities in Latvia) participated in the study; 4750 responses were suitable for data analysis. The final sample included nurses, nurse assistants, physicians and residents, physician assistants, and other licenced medical professionals such as physiotherapists, laboratory technicians, and radiology staff (see Table 1 for details). Responses from 392 participants were excluded due to incomplete or otherwise not valid questionnaires.

Although Latvia maintains registries of healthcare professionals, up-to-date counts of actively employed hospital staff is lacking, preventing precise response rate calculations. Based on 2017 national estimates of 48,900 health and social care professionals, of whom 56.6% worked in hospital settings, our analytic sample represents approximately 17% of Latvia’s estimated hospital workforce (Schneider et al., 2022).

### 2.2. Measurement

#### 2.2.1. Demographic and Health-Related Variables

Demographic characteristics collected included age, gender, occupation, medical specialty, number of workplaces, weekly working hours, presence of chronic illnesses, self-evaluation of overall health, days missed at work due to illness, days worked while ill (presenteeism), smoking frequency, and alcohol or drug use. Self-rated health was assessed with the item “How would you rate your current state of health?” This was a mandatory, single-choice question with five response options: Good, Fairly good, Average, Fairly poor, and Poor. Chronic illness was measured with the question “Do you have any chronic health conditions?” Respondents could select multiple answers from a predefined checklist of 14 diagnostic categories (e.g., cardiovascular, neurological, dermatological conditions) and had the option to enter additional conditions under “Other illness.”

#### 2.2.2. The Copenhagen Psychosocial Questionnaire

The Copenhagen Psychosocial Questionnaire (COPSOQ) III (Burr et al., 2019) is one of the most comprehensive instruments for measuring psychosocial risk factors in the workplace. Its broad coverage of psychosocial domains relevant to employee well-being enables the assessment of all major theoretical models related to psychosocial work environments. The COPSOQ was developed for assessing psychosocial working conditions and promoting workplace health by a research team led by Tage S. Kristensen and Vilhelm Borg at the Danish National Research Centre for the Working Environment (1995–2007). The questionnaire consists of core items and optional additional items, which researchers can select based on the study’s aims and context.

Based on this, 52 items covering five domains and 26 dimensions were included in the final version used in this study (see Table 2). The response options vary by item type but are rated on a 5-point Likert scale. The dimensions of conflicts and offensive behaviours were included in the analyses at the domain level.

#### 2.2.3. The Burnout Assessment Tool

The Burnout Assessment Tool (BAT) (Schaufeli et al., 2020) was applied to assess burnout symptoms. The instrument aligns with the JD-R model’s conceptualization of strain. The BAT is a self-report questionnaire developed by KU Leuven as an alternative to the Maslach Burnout Inventory (MBI), reflecting a more recent theoretical understanding of burnout.

In this study, we used the core module of the BAT along with additional scales assessing secondary and atypical symptoms. The core module consists of four subscales: exhaustion (8 items), mental distancing (5 items), emotional impairment (5 items), and cognitive impairment (5 items). The additional scales include psychological distress (6 items) and psychosomatic complaints (5 items). Following the developers’ guidelines, a composite score of the core module scales was used as the burnout variable in the analysis (Schaufeli et al., 2020).

### 2.3. Statistical Analysis

A multi-method analytical strategy using IBM SPSS Statistics Version 26 was applied to address the research questions. To examine how sociodemographic and job-related characteristics are associated with burnout (RQ1), one-way ANOVAs were used to compare mean burnout scores across relevant categorical groups (i.e., employment duration, number of workplaces, working hours, self-rated health, and substance use). To identify key psychosocial factors associated with burnout (RQ2), Pearson correlation analyses were conducted, followed by multiple linear regression (backwards method) using psychosocial variables from the COPSOQ III. Variance inflation factors (VIFs) were used to assess multicollinearity. To examine extreme risk profiles, a binomial logistic regression was performed comparing healthcare workers in the highest and lowest quartiles of burnout. This model assessed which psychosocial and demographic factors are associated with being in the high versus low burnout quartile, and model performance was evaluated using Nagelkerke R^2^ and classification accuracy (AUC). To assess whether protective workplace factors moderate the relationship between work overload and burnout (RQ3), a series of moderation analyses were conducted using the PROCESS macro for SPSS (Model 1). Each model tested the interaction between work overload and a single protective factor (i.e., support, recognition, trust) with mean-centring and bootstrapped confidence intervals. Analyses used 5000 bias-corrected bootstrapped samples to generate 95% confidence intervals. Where significant interactions were found, simple slopes analyses were conducted to interpret the direction and magnitude of moderation effects.

## 3. Results

### 3.1. Demographic and Work-Related Factors Associated with Burnout

Burnout levels differed significantly by self-rated health, presenteeism, working hours, absenteeism, and substance use (Table 3). Participants reporting very poor health had the highest burnout scores, while those in very good health had the lowest. Working while ill for 1–10 days, being absent due to illness for more than 20 days, working more than 60 h per week, and daily use of tobacco, alcohol, or non-prescribed psychotropic medication were all associated with higher burnout. No significant differences were observed by gender or duration of employment.

To examine the associations between demographic, health, and work-related variables and burnout while accounting for other variables, we conducted a multiple linear regression analysis (Table 4). The overall model was significant, F(11, 4127) = 140.68, *p* < 0.001, explaining 27.3% of the variance in burnout (R^2^ = 0.273, adjusted R^2^ = 0.271).

Higher burnout scores were most strongly associated with poorer self-rated health, more frequent presenteeism, use of non-prescribed psychotropic medication, more frequent alcohol, and younger age. Working more than 50 h per week was also significantly associated with higher burnout. Gender, number of workplaces, and tobacco/nicotine use were not significantly associated with burnout (*p* > 0.05) when other variables were considered.

### 3.2. Multivariate Analyses of the Factors Associated with Burnout

#### 3.2.1. Key Psychosocial Factors Associated with Healthcare Workers’ Burnout

Before conducting the hierarchical regression, Pearson correlations were computed to examine associations between burnout and psychosocial work factors (see Supplementary Table S1). As expected, burnout showed strong positive correlations with work–life interference (r = 0.53, *p* < 0.001), emotional demands (r = 0.44, *p* < 0.001), role conflict (r = 0.43, *p* < 0.001), and aggression at work (r = 0.39, *p* < 0.001). Conversely, burnout was strongly and negatively correlated with job satisfaction (excluding pay) (r = –0.46, *p* < 0.001), sense of belonging (r = –0.44, *p* < 0.001), organizational justice (r = –0.39, *p* < 0.001), predictability (r = –0.35, *p* < 0.001), and recognition (r = –0.34, *p* < 0.001). Other protective resources, including support from supervisors and colleagues, horizontal and vertical trust, and resources for quality care, were also significantly negatively correlated with burnout. To identify the psychosocial factors most strongly associated with burnout while accounting for background differences among staff, a two-step covariate-adjusted multiple linear regression analysis was conducted.

In Step 1, five demographic and job-related covariates were entered: respondent age, gender, years in healthcare, number of workplaces, and weekly working hours. This model was significant, F(5, 4133) = 41.66, *p* < 0.001, but explained only 4.8% of the variance in burnout scores (R^2^ = 0.048, adjusted R^2^ = 0.047).

In Step 2, 15 statistically and theoretically relevant psychosocial variables were added. The full model was significant, F(15, 4123) = 298.86, *p* < 0.001, and substantially improved explanatory power, increasing explained variance to 44.8% (R^2^ = 0.448, adjusted R^2^ = 0.445), representing an additional 40.0% of variance explained compared to Step 1 (ΔR^2^ = 0.400, *p* < 0.001).

The strongest correlates of burnout were work–life interference (β = −0.247, *p* < 0.001), emotional demands (β = −0.144, *p* < 0.001), and job satisfaction excluding pay (β = 0.203, *p* < 0.001). Other significant correlates included exposure to aggression (β = −0.096, *p* < 0.001), time pressure (β = −0.124, *p* < 0.001), sense of belonging (β = 0.096, *p* < 0.001), and resources for quality care (β = 0.070, *p* < 0.001). Among the demographic variables, younger age, more years in healthcare, and longer working hours were associated with higher burnout. No signs of multicollinearity were detected (all VIFs < 1.8) (see Table 5).

Several psychosocial variables (support from supervisors, support from colleagues, supervisor evaluation, recognition, predictability, vertical trust, organizational justice, and role conflict) and some covariates (e.g., age and weekly working hours) that were significantly correlated with burnout at the bivariate level were no longer significant in the covariate-adjusted multivariate model. This suggests that their apparent associations with burnout were largely accounted for by stronger overlapping factors such as work–life interference, emotional demands, and job satisfaction.

#### 3.2.2. Extreme Burnout Risk Profiles

To better understand extreme risk profiles, an exploratory logistic regression model comparing healthcare workers in the top and bottom quartiles of burnout was conducted (*n* = 1182 per group) (see Table 6). Despite the more stringent threshold, the results closely mirrored previous findings. The model demonstrated excellent discrimination (AUC = 0.946) and fit (Nagelkerke R^2^ = 0.729), correctly classifying 87.5% of cases. Work–life interference remained the strongest factor associated with high burnout (OR = 2.56), followed by emotional demands, time pressure, and role conflict. Protective factors associated with lower burnout included job satisfaction (excluding pay), sense of belonging, and horizontal trust, reinforcing the importance of social cohesion and meaningful work.

Demographic comparisons indicated that individuals in the high-burnout group tended to be younger, worked longer hours, and were more likely to hold multiple jobs (see Table 7). While number of workplaces and gender were not significantly associated with burnout in the overall regression analysis, both were more frequently observed in the highest burnout quartile: women and those holding multiple jobs were overrepresented among individuals with the highest levels of burnout. This pattern suggests that group differences related to gender and number of workplaces may be more pronounced at extreme levels of burnout, even if not evident in the overall sample.

### 3.3. Mediation Analyses: Work–Life Interference as a Mechanism Associated with Job Demands and Burnout

Work–life interference had strongest association with burnout in the present study. To further examine the role of work–life interference as a potential mechanism linking excessive job demands to burnout, two mediation analyses were conducted using Model 4 of the PROCESS macro (Hayes, 2022). In each model, work–life interference was specified as the mediator between either work overload or emotional demands, and burnout. All variables were mean-centred, and 5000 bootstrapped samples were used to estimate indirect effects. Both models tested whether work–life interference mediates the relationship between job demands (work overload or emotional demands) and burnout among healthcare professionals.

In the first model, work overload significantly associated with greater work–life interference, which in turn is associated with higher burnout. The direct effect of work overload on burnout remained significant after accounting for the mediator, indicating partial mediation. The indirect effect was statistically significant (see Table 8).

In the second model, emotional demands significantly associated with greater work–life interference, which was again associated with higher burnout. The direct effect of emotional demands on burnout remained significant, and the indirect effect was also significant, indicating partial mediation (see Table 8).

These findings suggest that work overload and emotional demands are associated with an increased likelihood of burnout both directly and indirectly through their association with the conflict between work and private life. The presence of a significant direct effect alongside the mediated pathway suggests that work–life interference only partially accounts for the relationship between job demands and burnout. This implies that additional mechanisms, not captured in the current model, also be associated with burnout in the context of high workload and emotional demands.

### 3.4. Moderation Analyses: Psychosocial and Organizational Moderators of the Association Between Work Overload and Burnout

To examine whether key organizational and psychosocial resources moderate the association between work overload and burnout, a series of moderation analyses were conducted using Model 1 of the PROCESS macro (Hayes, 2022). All continuous variables were mean-centred prior to analysis. The independent variable in each model was work overload, the dependent variable was burnout, and each model included a single moderator. Interaction terms were computed as the product of the centred independent variable and moderator. The significance of the interaction term indicates moderation.

#### 3.4.1. Buffering Effects of Psychosocial Resources

Several models showed significant interactions in the expected direction, supporting the buffering hypothesis (see Table 9). These included support from colleagues, a sense of belonging, supervisor support, supervisor evaluation, resources for quality work, and recognition. In each case, both workload and the moderator were independently associated with burnout, and the interaction term was significant. Simple slopes analyses confirmed that the positive association between workload and burnout was attenuated when these resources were high, indicating that these psychosocial factors were associated with an attenuation of the relationship between excessive workload and burnout.

Although the effect sizes were modest, the consistency of the pattern across these factors suggests that social support, leadership quality, and resource adequacy are associated with a weaker relationship between workload and burnout.

#### 3.4.2. Non-Significant Moderation Results

Horizontal trust showed a main effect, being negatively associated with burnout, but did not significantly moderate the association between work overload and burnout. This suggests that while trust among peers is important for well-being, it may not be associated with an attenuation of the relationship between workload and burnout in this context (see Table 9).

#### 3.4.3. Amplifying Effects and Unexpected Findings

In contrast to the buffering hypothesis, several factors typically considered protective, namely, perceived influence, meaning and development, organizational justice, and role conflict were associated with a stronger relationship between workload and burnout when present at high levels (see Table 9). In these models, significant interaction terms indicated that the association between workload and burnout was amplified under conditions of high perceived influence, meaningful work, organizational justice, or role conflict.

For perceived influence, workload alone was not independently associated with burnout; however, the interaction showed that burnout levels were higher under high workload when influence was also high. This pattern may reflect greater emotional investment or responsibility among employees with more autonomy, potentially resulting in higher strain under excessive demands. Similarly, high levels of meaning and development amplified the association between workload and burnout, which is consistent with the concept of self-endangering behaviour where overcommitment to meaningful work may be linked to emotional exhaustion.

Organizational justice, while generally protective, also showed an amplifying association: high perceived fairness was linked to a stronger relationship between workload and burnout. One possible interpretation is that unmet expectations for equity may contribute to frustration in under-resourced environments. Role conflict likewise amplified the workload–burnout association, suggesting that conflicting demands or expectations (e.g., incompatible tasks or ethical dilemmas) may further intensify strain and exhaustion when workloads are high.

## 4. Discussion

This study examined associations between sociodemographic factors, psychosocial work conditions, and burnout among Latvian hospital-based healthcare professionals during the post-acute phase of the COVID-19 pandemic. Our findings indicate that burnout in this sample was primarily associated with psychosocial and organizational factors, while demographic variables showed less significant associations. Among psychosocial factors, work–life conflict, emotional demands, and time pressure showed the strongest associations with burnout. These patterns are consistent with cross-national findings among hospital staff during the pandemic, where heightened emotional demands, uncertainty, and disrupted work–life boundaries were strongly associated with burnout (Koutsouri et al., 2023). In our context, extended shifts, multiple employments, and staffing shortages, conditions intensified by pandemic-related disruptions, likely contributed to these dynamics. Psychosocial resources, such as supervisor support, sense of belonging, and organizational justice, were also associated with lower burnout, although their buffering effects weakened under chronic overload.

Unexpectedly, several resources typically considered protective, including autonomy, meaning, and influence, were linked to higher burnout when job demands were high, suggesting the role of self-endangering behaviours. This highlights a structural paradox in hospital care: the very values that sustain healthcare professionals, such as commitment to patients, professional pride, and purpose, can become sources of strain when institutional support is lacking. These findings are interpreted below in relation to our research questions and hypotheses and are discussed within the framework of established theoretical models of burnout.

### 4.1. Sociodemographic and Job-Related Characteristics Associated with Burnout

To address our first research question, we examined whether sociodemographic and job-related characteristics were associated with burnout. Our findings align with global evidence indicating that structural and behavioural work factors explain the majority of variance in burnout, while demographic characteristics contribute relatively little (Marques-Pinto et al., 2021; Meredith et al., 2022). In our sample, no significant associations were found between burnout and either gender or tenure, and age demonstrated only a modest inverse relationship with burnout, echoing findings from European and Canadian cohorts in which older clinicians tend to report slightly lower exhaustion once workload is controlled (Adam et al., 2018; Gómez-Urquiza et al., 2017; Marchand et al., 2018).

The clearest differentiators were self-reported health status and recovery-related work habits. Clinicians who rated their health as poor, worked while sick, or routinely exceeded 50–60 working hours per week reported the highest burnout. These results extend prior COVID-19-era studies showing that presenteeism and excessive overtime are strongly associated with higher burnout and they may act as “accelerants” in the burnout process by compressing recovery windows (Kinman & Grant, 2021). In the Latvian context, where multiple employments are common due to relatively low wages, staffing shortages, and restrictive sick leave policies, the opportunity cost of rest is unusually high, reinforcing a culture of chronic overextension. This burden is particularly pronounced among nurses.

Substance use (daily tobacco, frequent alcohol, non-prescribed psychotropics) was positively associated with burnout, consistent with its potential role as a maladaptive coping strategy rather than a primary cause. This observation supports qualitative reports of clinicians “self-medicating” in the absence of formal psychological support (Dūdiņa & Martinsone, 2024). Similar links have been documented in several European studies and US cohorts since the pandemic surge (Alexandrova-Karamanova et al., 2016), suggesting a cross-cultural pattern.

Together, these patterns suggest that in Latvia’s healthcare system, burnout may be best understood through a recovery deficit lens: poor health, presenteeism, long hours, and psychosocial demands are linked to reduced capacity for resource replenishment.

### 4.2. Psychosocial Factors Associated with Burnout

In addressing the second research question, psychosocial work factors, particularly work–life conflict, emotional demands, and time pressure, showed the strongest associations with burnout among Latvian hospital staff. Protective resources such as peer cohesion, fair procedures, and supervisor support were linked to lower burnout, though these effects tended to weaken in chronically overloaded systems. This aligns with Scandinavian and pan-European research showing that relational resources buffer strain only when workloads remain manageable (Christiansen et al., 2024; Leineweber et al., 2014; Antolí-Jover et al., 2024). In Latvia, structural challenges such as multiple jobs and extended shifts, exacerbated by the COVID-19 pandemic, likely intensified work–life conflict and time pressure.

Work–life conflict emerged as the strongest psychosocial correlate of burnout, consistent with European nursing studies across both pre- and post-pandemic contexts. As predicted by Conservation of Resources (COR) theory, intrusion of work into personal time impairs recovery and heightens exhaustion risk (Hobfoll, 1989). Emotional labour also showed a robust association with burnout, underscoring the toll of sustained empathy and exposure to suffering (Leiter & Maslach, 2003). Despite this, emotional labour remains under-addressed in workforce policy, often framed as an individual rather than organizational issue (Office of the U.S. Surgeon General, 2022). Time pressure, the third most impactful factor, was linked to fragmented attention, emotional dysregulation, and self-endangering behaviours such as skipped breaks (Eder & Meyer, 2023). Notably, our moderation analyses suggest that under high workload, resources such as autonomy and meaning may paradoxically associate with higher burnout, aligning with the self-endangerment model.

Among protective psychosocial resources, a sense of belonging and horizontal trust were linked to lower burnout, reinforcing evidence that peer support can buffer strain in high-demand environments (Simms et al., 2023). While formal organizational supports (e.g., supervisor support, recognition, procedural justice) also showed protective associations, they lost significance in multivariate models, likely reflecting their diminished impact in resource-constrained settings. According to COR theory, resources must be perceived as real and attainable to offer protection; when unmet, they may generate additional strain. Cross-national findings highlight the context-dependent nature of leadership efficacy: some studies report null effects for supervisor support (Christiansen et al., 2024). Longitudinal studies are needed to further clarify these dynamics. To explore how psychosocial resources interact with burnout under varying levels of strain, we next turn to moderation analyses in Section 4.3.

### 4.3. Buffering and Amplifying Roles of Psychosocial Factors in Burnout

#### 4.3.1. Psychosocial Resources Associated with Lower Burnout Under Conditions of High Job Demand

Among the moderators tested, resources that ensured quality care showed the strongest buffering effect, weakening the association between work overload and burnout. This finding underscores the importance of structural conditions, such as adequate time, tools, and team capacity, that enable healthcare professionals to deliver care effectively and uphold clinical standards; when these are lacking, lower perceived care quality and greater emotional strain are reported.

Social resources, including support from colleagues and supervisors and positive leadership evaluations, also demonstrated significant, though modest, moderating effects. However, overall levels of collegial and supervisor support were relatively low in our sample, highlighting the potential for workplace strategies to strengthen team communication and promote emotionally attuned supervisory contact. Leadership quality further emerged as an important contextual factor: competent, emotionally responsive leadership was associated with lower burnout levels under high job demand, potentially functioning as a source of social support and structural stability, which may be linked to reduced uncertainty, reinforcement of professional standards, and lower stress associated with inconsistent management. However, the effect sizes were modest, and findings from Swedish physicians (Christiansen et al., 2024) similarly suggest that under chronic strain, even high-quality leadership may be insufficient to fully buffer the impact of excessive workload.

Taken together, these results support the buffering hypothesis of the JD-R model but also indicate that protective effects are limited under persistent overload, leading us to examine whether some resources might instead intensify burnout risk.

#### 4.3.2. Psychosocial Resources Associated with Higher Burnout Under Conditions of High Job Demand

Contrary to these buffering effects and the assumptions of the JD-R and JDCS models, several factors typically viewed as resources, including perceived influence, meaningful work, professional development, and organizational justice, were associated with a stronger positive relationship between workload and burnout. Clinicians reporting high influence, strong intrinsic motivation, or perceptions of fairness experienced greater increases in burnout under rising workload, a counterintuitive pattern that challenges traditional theoretical expectations.

These findings align with emerging evidence on self-endangering behaviours, where mission-driven professionals internalize systemic pressures and push beyond safe limits (Eder & Meyer, 2023). In under-resourced environments, autonomy, ethical commitment, and professional meaning can heighten responsibility without providing adequate means to meet elevated standards. As a result, resources that are typically protective may transform into additional demands, amplifying the risk of emotional exhaustion.

### 4.4. Theoretical Contributions

Our findings refine established models of occupational stress and burnout by revealing greater complexity in the relationships between job demands, resources, and burnout. Consistent with the JD-R model and COR theory, high emotional demands, quantitative overload, and work–life conflict were most strongly associated with higher burnout, illustrating the cumulative impact of resource loss and insufficient recovery.

However, our results also challenge the classic JD-R assumption that job resources always buffer the effects of demands. Under chronic overload, resources such as professional commitment, autonomy, and meaning were associated with higher burnout, paralleling recent evidence on self-endangering behaviours among mission-driven professionals (Eder & Meyer, 2023). These patterns, observed in our sample, reflect a structural paradox: in understaffed, high-pressure settings, acts of self-sacrifice and overextension may be normalized, despite their psychological cost.

By integrating these moral and systemic dynamics, our findings extend current theories and underscore the need for structural interventions that address not only workload but also organizational norms that valorize overcommitment. This perspective is crucial for designing effective prevention strategies and supporting the well-being of healthcare professionals.

### 4.5. Implications for Policy and Practice

Our findings emphasize that reducing burnout among hospital staff requires interventions targeting not just individuals, but also the structural and systemic drivers in the psychosocial work environment. Relying solely on resilience training or peer support is insufficient without accompanying organizational change. To address chronic understaffing and excessive workload, priority should be given to staffing reforms, protected rest periods, workload monitoring, and workflow streamlining to reduce administrative burden and role conflict. Protecting work–life boundaries through predictable scheduling, flexible leave, and integration of work–life conflict metrics into institutional audits is also essential.

In addition, healthcare organizations should formally recognize emotional labour as an occupational demand and provide structured reflective practices (such as Schwartz Rounds), supervision, and leadership training in emotional intelligence and psychological safety. Fostering peer support, participatory leadership, career development, and team cohesion can further strengthen psychosocial resources. Investments in leadership capacity, including training, accountability, and promoting procedural justice, are critical, as is ensuring confidential access to mental healthcare, psychoeducation, and anti-stigma efforts.

Finally, our results caution against overreliance on traditional protective factors like autonomy or professional meaning in contexts of chronic overload, where these resources may paradoxically intensify burnout. Effective interventions must consider context and ensure that structural resources, such as sufficient staffing and recovery opportunities, are in place for relational and motivational resources to provide real benefit.

### 4.6. Study Strengths, Limitations, and Directions for Future Research

A key strength of this study is the large and diverse sample of healthcare professionals drawn from 30 of the 37 hospitals in Latvia, providing broad representation across different institutional contexts. The use of validated psychosocial measures and advanced statistical techniques, including multivariable modelling and moderation analyses, enabled an in-depth examination of associations between psychosocial work factors and burnout. Together, these features enhance the study’s methodological rigour and support the robustness of its findings.

Several limitations should be noted. First, the cross-sectional design precludes causal inference, and reliance on self-reported data may introduce measurement bias. As data collection occurred during the post-acute phase of the COVID-19 pandemic, some findings may reflect pandemic-specific stressors, which could limit their generalizability to non-crisis periods. Furthermore, the study did not differentiate factors associated with burnout across specific healthcare professions (e.g., physicians versus nurses), which may mask important occupational differences. Additionally, as participation in the study was voluntary, self-selection bias may have affected the results, particularly if individuals experiencing higher or lower distress were more or less likely to respond.

Future studies should adopt longitudinal designs to examine burnout trajectories over time and to clarify causal pathways between job demands, resources, and burnout. Research should also evaluate whether organizational interventions (e.g., staffing reforms, leadership training) are associated with changes in mediating and moderating mechanisms, such as work–life conflict and team support. Subgroup analyses are needed to explore differential vulnerabilities based on profession, gender, career stage, or family responsibilities, which may inform more targeted prevention strategies.

## 5. Conclusions

This study contributes to a growing body of evidence indicating that burnout in healthcare is strongly associated with structural and psychosocial conditions rather than individual vulnerability. Our findings support and refine established models such as the Job Demands–Resources (JD-R) and Conservation of Resources (COR) frameworks. In particular, they highlight how emotional demands, quantitative overload, and work–life conflict showed cumulative associations with emotional exhaustion and disengagement.

Crucially, our results challenge the universal buffering assumption within the JD-R model. Under chronic overload, certain job resources, including autonomy, professional meaning, and perceived fairness, were associated with higher burnout. This paradox aligns with emerging evidence on self-endangering work behaviour, in which intrinsically motivated professionals report overextending themselves in response to institutional pressures and internalized ethical commitments. By integrating moral and systemic dimensions into the understanding of burnout, our study underscores that even traditionally protective factors may be associated with higher burnout risks in resource-depleted environments.

Burnout should therefore be understood not as an individual failure of resilience but as closely associated with systemic misalignments that demand self-sacrifice without enabling recovery. Structural interventions must target both excessive demands and the organizational norms that perpetuate overcommitment.

Future research should explore when and under what conditions resources function as buffers versus amplifiers, and advance context-sensitive models of occupational health that account for the complex interplay between job demands, resources, and systemic pressures.

## Figures and Tables

**Table 1:** Demographic Characteristics of the Study Sample.

Professional Category	*n*	% of Sample
Nurses	1568	33%
Nurse assistants	950	20%
Physicians and residents	855	18%
Physician assistants	333	7%
Other licenced medical professionals ^1^	997	21%
Total analyzed sample	4750	100%

^1^ Other licenced medical professionals include physiotherapists, laboratory technicians, and radiology staff.

**Table 2:** COPSOQ III Domains and Dimensions Included in the Questionnaire.

Domain	Dimension
Demands at Work	Work Pace
	Work Overload
	Emotional Demands
Work Organization and Job Contents	Influence at Work
	Possibilities for Development
	Control
	Meaning of Work
Interpersonal Relations and Leadership	Predictability
	Recognition
	Quality of Leadership
	Social Support from Supervisor
	Social Support from Colleagues
	Sense of Community at Work
Work Individual Interface	Commitment to the Workplace
	Quality of Work
	Job Satisfaction
	Work Life Conflict
Social Capital	Vertical Trust
	Horizontal Trust
	Organizational Justice
Conflicts and Offensive Behaviour	Gossip and Slander
	Conflicts and Quarrels
	Unpleasant Teasing
	Cyber Bullying
	Sexual Harassment
	Threats of Violence
	Physical Violence
	Bullying

**Table 3:** ANOVA Results for Burnout Across Demographic and Work-Related Variables.

Category	*n*	*%*	*F*	*p*
Duration of Employment			0.98	0.632
0–2 years	283	5.94%		
3–5 years	548	11.5%		
6–10 years	547	11.5%		
11–15 years	439	9.23%		
16–20 years	540	11.35%		
21–30 years	391	8.22%		
31+ years	1391	29.25%		
Number of Current Workplaces			1.12	0.050
1 workplace	2993	62.9%		
2 workplaces	1268	26.6%		
3 workplaces	328	6.9%		
4+ workplaces	167	3.5%		
Working Hours per Week			1.40	<0.001
<40 h	2003	42.1%		
40–50 h	1666	35%		
51–60 h	659	13.8%		
>60 h	428	9%		
Self-Rated Health			3.20	<0.001
Very good	1120	23.5%		
Good	1611	33.8%		
Average	1778	37.4%		
Poor	226	4.7%		
Very poor	21	0.4%		
Days Absent from Work Due to Illness			1.20	0.002
0 days	1874	39.4%		
1–10 days	1726	36.3%		
11–20 days	630	13.2%		
More than 20 days	526	11.1%		
Worked While Sick			3.02	<0.001
0 days	2677	56.3%		
1–10 days	1649	34.6%		
11–20 days	219	4.6%		
More than 20 days	211	4.4%		
Tobacco or Nicotine Use			1.12	0.039
Yes, daily	921	19.4%		
Yes, several times a week	149	3.1%		
Yes, several times a month	92	1.9%		
Once a month or less	84	1.8%		
A few times a year	137	2.9%		
No	3373	70.9%		
Use of Non-Prescribed Psychotropic Meds			2.94	<0.001
Yes, daily	22	0.5%		
Yes, several times a week	40	0.8%		
Yes, several times a month	51	1.1%		
Once a month or less	59	1.2%		
A few times a year	141	3%		
No	4443	93.4%		
Frequency of Alcohol Use			1.68	<0.001
Daily	25	0.5%		
Several times a week	290	6.1%		
Several times a month	1055	22.2%		
Once a month or less	1152	24.2%		
A few times a year	1285	27.0%		
I do not use alcohol	949	19.9%		
Gender			1.60	0.050
Female	558	11.7%		
Male	4198	88.3%		

Note. Values represent counts (*n*), percentages (%), and results of one-way ANOVA tests (*F*) for associations between demographic and occupational characteristics and burnout levels.

**Table 4:** Regression Coefficients for Demographic Factors Associated with Burnout in Healthcare Workers.

Model Summary	*R* ^2^	*Adjusted R* ^2^	*F*	*df*	*p*
Full Model	0.273	0.271	140.68	11, 4127	<0.001
Variable		*B*	*β*	*t*	*p*
Constant		2.006		22.940	<0.001
Respondent Age		−0.006	−0.159	−9.297	<0.001
Gender		0.044	0.028	2.055	0.040
Years Worked in Healthcare		0.020	0.084	5.057	<0.001
Number of Current Workplaces		0.004	0.006	0.375	0.708
Average Weekly Working Hours		0.021	0.039	2.643	0.008
Self-Rated Health		0.168	0.299	20.747	<0.001
Days Absent from Work Due to Illness		0.020	0.040	2.935	0.003
Worked While Sick		0.123	0.188	13.031	<0.001
Tobacco or Nicotine Use		−0.001	−0.003	−0.228	0.820
Use of Non-Prescribed Psychotropic Medications		−0.112	−0.141	−10.434	<0.001
Alcohol Use Frequency		−0.053	−0.128	−9.254	<0.001

Note. *B* = unstandardized regression coefficient; *β* = standardized regression coefficient.

**Table 5:** Hierarchical Linear Regression of Factors Associated with Burnout.

Model Summary	*R* ^2^	*Adjusted R* ^2^	*ΔR* ^2^	*F*	*df*	*p*
Step 1: Covariates	0.048	0.047	—	41.66	5, 4133	<0.001
Step 2: Full Model	0.448	0.445	0.400	298.86	15, 4123	<0.001
Predictor		*B*	*SE B*	*β*	*t*	*p*
Step 1: Covariates						
Age		−0.008	0.001	−0.213	−11.16	<0.001
Gender		0.049	0.025	0.031	2.01	0.045
Years in healthcare		0.035	0.005	0.144	7.53	<0.001
Number of workplaces		0.005	0.011	0.008	0.47	0.639
Weekly working hours		0.056	0.009	0.105	6.16	<0.001
Step 2: Psychosocial predictors						
Job satisfaction (excluding pay)		0.15	0.012	0.203	12.25	<0.001
Time pressure		−0.056	0.006	−0.124	−9.07	<0.001
Work pace		0.031	0.007	0.057	4.51	<0.001
Resources for quality care		0.053	0.012	0.07	4.56	<0.001
Work–life interference		−0.1	0.006	−0.247	−16.08	<0.001
Emotional demands		−0.074	0.008	−0.144	−9.63	<0.001
Predictability		0.008	0.009	0.015	0.92	0.356
Role conflict		−0.002	0.008	−0.005	−0.29	0.775
Recognition		0.004	0.01	0.008	0.43	0.668
Support from supervisor		−0.011	0.007	−0.025	−1.45	0.146
Supervisor evaluation		0.005	0.011	0.01	0.49	0.628
Support from colleagues		0.008	0.008	0.014	0.98	0.326
Horizontal trust		−0.033	0.011	−0.039	−3.03	0.002
Vertical trust		0.018	0.013	0.027	1.43	0.153
Organizational justice		−0.017	0.013	−0.03	−1.32	0.186
Violence/aggression		−0.106	0.016	−0.096	−6.55	<0.001
Sense of belonging		0.06	0.011	0.096	5.65	<0.001

Note. *B* = unstandardized coefficient; *SE B* = standard error of *B*; *β* = standardized coefficient. All variables were entered simultaneously within each step.

**Table 6:** Logistic Regression Predicting High Burnout Group Membership.

Predictor	*B*	*SE B*	*Wald’s χ* ^2^	*p*	*OR*
Work–Life Interference	0.941	0.071	173.74	<0.001	2.56
Emotional Demands	0.684	0.082	68.97	<0.001	1.98
Job Satisfaction (Excluding Pay)	−0.97	0.13	55.68	<0.001	2.64
Sense of Belonging	−0.775	0.105	54.06	<0.001	2.17
Lack of Time to Complete Work	0.474	0.068	48.03	<0.001	1.61
Working at Fast Pace	0.29	0.08	13.09	<0.001	1.34
Clear Work Goals (Role Clarity)	0.355	0.1	12.51	<0.001	1.43
Perceived Work Quality	−0.449	0.129	10.91	<0.001	1.57
Horizontal Trust	−0.324	0.121	7.19	<0.001	0.72

Note. *OR* = odds ratio. All variables retained in step 12 of backwards elimination. Overall model classification accuracy = 87.5%.

**Table 7:** Demographic Characteristics in High and Low Burnout Groups.

Demographic Characteristics	High Burnout	Low Burnout	*χ*^2^ (*df*)	*p*
	*n* (*%*)	*n* (*%*)		
Gender				
Male	130 (10.9%)	169 (14.1%)	5.70 (1)	0.017
Female	1062 (89.1%)	1026 (85.9%)		
Years in healthcare				
0–2	49 (5.0%)	113 (10.3%)	29.46 (6)	<0.001
3–5	122 (12.5%)	161 (14.7%)		
6–10	139 (14.3%)	140 (12.8%)		
11–15	127 (13.0%)	102 (9.3%)		
16–20	131 (13.4%)	139 (12.7%)		
21–30	98 (10.1%)	91 (8.3%)		
31+	308 (31.6%)	348 (31.8%)		
Number of workplaces				
1	687 (57.6%)	818 (68.5%)	37.90 (3)	<0.001
2	349 (29.3%)	278 (23.3%)		
3	113 (9.5%)	57 (4.8%)		
4+	43 (3.6%)	42 (3.5%)		
Weekly hours (last 3 months)				
<40	408 (34.2%)	623 (52.1%)	103.11 (3)	<0.001
40–50	432 (36.2%)	393 (32.9%)		
51–60	211 (17.7%)	105 (8.8%)		
>60	141 (11.8%)	74 (6.2%)		

Note. *N* = 1182 for each burnout group. Values represent frequencies and row percentages. *χ*^2^(*df*) = chi-square statistic with degrees of freedom. 25% threshold used.

**Table 8:** Mediation Analyses: Work–Life Interference as a Mediator of the Association Between Job Demands and Burnout.

Model	Path	*B*	*SE*	*T*	*p*	*95% CI*
1	Work Overload → Work–Life Interference	−0.4613	0.0146	−31.64	<0.001	[−0.4899, −0.4327]
	Work–Life Interference → Burnout	−0.1843	0.0054	−33.83	<0.001	[−0.1950, −0.1736]
	Direct Effect	−0.0828	0.0060	−13.75	<0.001	[−0.0947, −0.0710]
	Indirect Effect	—	—	—	—	(Significant, bootstrapped)
2	Emotional Demands → Work–Life Interference	−0.6275	0.0162	−38.74	<0.001	[−0.6593, −0.5958]
	Work–Life Interference → Burnout	−0.1681	0.0056	−29.92	<0.001	[−0.1791, −0.1571]
	Direct Effect	−0.1242	0.0072	−17.25	<0.001	[−0.1383, −0.1101]
	Indirect Effect	−0.1055	0.0052	—	—	[−0.1155, −0.0956]

Note. Coefficients are unstandardized. All variables were mean-centred. Indirect effect derived from 5000 bootstrapped samples. *CI* = confidence interval. *N* = 4756.

**Table 9:** Moderation Analysis: Psychosocial and Organizational Factors as Moderators of the Relationship Between Work Overload and Burnout.

Moderator	Model *R^2^*	*B* Work Overload	B Moderator	B Interaction	Interaction *p*	*95% CI*	Interpretation
Colleague Support	0.186	0.116 *	−0.187 *	−0.021 *	0.001	[−0.0324, −0.0086]	Buffering
Sense of Belonging	0.281	0.056 *	−0.364 *	−0.037 *	<0.001	[−0.0503, −0.0240]	Buffering
Horizontal Trust	0.164	0.158 *	−0.148 *	0.001	0.895	[−0.0174, 0.0199]	No moderation
Supervisor Support	0.192	0.120 *	−0.152 *	−0.015 *	0.002	[−0.0248, −0.0059]	Buffering
Supervisor Evaluation	0.218	0.097 *	−0.222 *	−0.021 *	<0.001	[−0.0324, −0.0097]	Buffering
Resources for Quality Work	0.227	0.060 *	−0.287 *	−0.032 *	<0.001	[−0.0448, −0.0195]	Buffering
Perceived Influence	0.168	Ns	−0.242 *	0.045 *	<0.001	[0.0310–0.0579]	Amplification
Meaning & Development	0.187	0.094 *	−0.236 *	0.032 *	< 0.001	[0.0190–0.0443]	Amplification
Role Conflict	0.242	0.204 *	0.263 *	0.026 *	<0.001	[0.0147–0.0367]	Amplification
Recognition	0.227	0.094 *	−0.234 *	−0.022 *	<0.001	[−0.0331, −0.0103]	Buffering
Organizational Justice	0.227	0.052 *	−0.293 *	0.034 *	<0.001	[0.0218–0.0469]	Amplification

Note. All continuous variables were mean-centred. Each model tested a separate moderation using the PROCESS macro for SPSS (Model 1), with burnout as the dependent variable. The interaction term reflects the product of work overload and the moderator. *p* values: *p* < 0.05 = *. *CI* = confidence interval. *N* = 4756.

## Data Availability

The raw data supporting the conclusions of this article will be made available by the authors upon request.

## Supplementary Materials

The following supporting information can be downloaded at: https://www.mdpi.com/article/10.3390/ejihpe15090186/s1, Table S1: Pearson correlations between burnout and psychosocial work factors.
