# Supplementary material for: Psychosocial Risks and Protective Factors for Healthcare Worker Burnout During the Post-Acute Phase of the COVID-19 Pandemic
Source: Eur J Investig Health Psychol Educ. 2025 Sep 16;15(9):186. doi: 10.3390/ejihpe15090186 (PMC12468918; doi:10.3390/ejihpe15090186)
Supplement: Supplementary file 1 [file ejihpe-15-00186-s001.zip › ejihpe-3803111-supplementary.pdf]

Supplementary materials

Table S1. Pearson correlations between burnout and psychosocial work factors.

Variable	M	SD	1	2	3	4	5	6	7	8	9	10	11	12	13	14	15	16	17	18	19	20
1. Burnout	1.69	.51	1.00																			
2. Satisfaction with Pay	2.83	1.06	-.17**																			
3. Satisfaction without Pay	2.36	.70	-.46**	.40**																		
4. Time Pressure	3.32	1.12	.37**	-.08**	.18**																	
5. Work Pace	2.25	.92	.13**	.04**	.11**	.28**																
6. Job Quality	2.43	.67	-.40**	-.25**	-.54**	.23**	.09**															
7. Work–Life Interference	3.23	1.23	.53**	.13**	.33**	-.42**	-.26**	.29**														
8. Emotional Demands	2.89	.96	.44**	.10**	.27**	-.34**	-.25**	.27**	-.49**													
9. Influence	3.14	.82	-.15**	-.20**	-.29**	.00	.13**	-.29**	.15**	.06**												
10. Opportunities and Meaning	2.41	.84	-.20**	-.27**	-.47**	-.03	-.01	-.37**	.07**	-.03	-.34**											
11. Predictability	2.56	.91	-.35**	-.22**	-.44**	.20**	.09**	-.49**	.27**	.25**	-.29**	-.37**										
12. Role Conflict	3.52	.96	.43**	.18**	.38**	-.35**	-.19**	.43**	-.42**	-.46**	.16**	.17**	.44**									
13. Recognition	2.56	.93	-.34**	-.33**	-.49**	.13**	.10**	-.49**	.27**	-.46**	-.38**	-.42**	-.57**	.43**								
14. Support from Supervisor	2.22	1.17	-.28**	-.19**	-.36**	.12**	.09**	-.37**	.25**	-.37**	-.28**	-.29**	-.47**	.37**	-.61**							
15. Supervisor Evaluation	2.49	.94	-.33**	-.25**	-.45**	.14**	.09**	-.45**	.27**	-.45**	-.29**	-.38**	-.58**	.43**	-.66**	-.72**						
16. Support from Colleagues	2.00	.93	-.26**	-.11**	-.45**	.12**	.06**	-.24**	.18**	-.37**	-.19**	-.24**	-.28**	.26**	-.37**	-.37**	-.35**					
17. Sense of Belonging	2.05	.80	-.44**	-.22**	-.45**	.20**	.09**	-.40**	.35**	-.49**	-.22**	-.31**	-.43**	.42**	-.49**	-.52**	-.56**	-.44**				
18. Horizontal Trust	3.34	.58	-.23**	-.08**	-.14**	.19**	.05**	-.18**	.19**	.21**	.00	-.07**	-.22**	.35**	-.18**	.52**	.56**	-.15**	-.23**			
19. Vertical Trust	2.29	.72	-.30**	-.22**	-.39**	.14**	.05**	-.44**	.21**	.20**	-.29**	-.38**	-.50**	.43**	-.60**	-.52**	-.60**	-.37**	-.45**	.20**		
20. Organizational Justice	2.70	.86	-.39**	-.31**	-.49**	.16**	.13**	-.50**	.32**	.35**	-.35**	-.39**	-.59**	.45**	-.71**	-.61**	-.71**	-.39**	-.50**	.21**	-.75**	
21. Aggression	1.35	.45	.39**	.13**	.27**	-.25**	-.13**	.25**	-.32**	-.35**	.10**	.12**	.31**	-.45**	.37**	.33**	.36**	.32**	.46**	-.31**	.37**	.39**

*p< 0.05; ** p< 0.01; *** p< 0.001. N = 4,756.
